# Effects of Load Carriage on Postural Sway and Relative Ground Reaction Forces in Special Police Officers: A Cross-Sectional Study

**DOI:** 10.3390/ijerph192416710

**Published:** 2022-12-13

**Authors:** Mario Kasović, Tomaš Vespalec, Lovro Štefan

**Affiliations:** 1 Department of General and Applied Kinesiology, Faculty of Kinesiology, University of Zagreb, 10 000 Zagreb, Croatia; 2 Department of Sport Motorics and Methodology in Kinanthropology, Faculty of Sports Studies, Masaryk University, 62 500 Brno, Czech Republic; 3 Department of Kinesiology, Faculty of Sports Studies, Masaryk University, 62 500 Brno, Czech Republic; 4 Department of Research and Examination (RECETOX), Faculty of Science, Masaryk University, 62 500 Brno, Czech Republic

**Keywords:** center of pressure, special police, gait movement, heavy load, changes

## Abstract

Although excessive load carriage results in biomechanical gait changes, little evidence has been provided regarding its impact on postural sway. Therefore, the main purpose of this study was to determine whether heavier loads have effects on changing foot stability and postural sway in special police officers. Thirty male special police officers (age = 40 ± 6 years, height = 180 ± 5 cm, weight = 89 ± 8 kg) were assessed in four conditions: (1) carrying no load, (2) carrying a 5 kg load, (3) carrying a 25 kg load, and (4) carrying a 45 kg load. Foot characteristics during standing were assessed with Zebris pedobarographic pressure platform. Heavier loads increased the center of pressure (COP) path length and average velocity, length of minor and major axis, and 95% confidence ellipse area, while a decrease in angle between Y and major axis was observed. Relative forces beneath the left forefoot and right backfoot regions decreased and an increase in relative forces beneath the left backfoot and right forefoot was observed. When carrying heavy loads, static foot parameters rapidly changed, especially in COP path length and average velocity.

## 1. Introduction

Although carrying excessive load is part of specific military training and operation protocols [1,2], it is associated with a few negative health-related outcomes, including the increased risk of lower limb injury [2] and a decrease in physical performance [3,4]. A historical perspective of military load shows an increase in load weight over time, which often leads to task inefficiencies due to different ergonomics and design of the load carried into combat [5]. Given the importance of optimal load carriage, which does not affect human posture and gait characteristics, studies have shown that special forces need to carry a relative load for tactical requirements between 45 and 57 kg (46–70% body weight) [6].

In order to compensate these heavy external loads, the bearer undergoes changes to their gait and posture [7]. Indeed, equipment consisting primarily of a rucksack can impede stability, balance and movement, making it more difficult to balance and stop or initiate movement [8,9]. This may produce greater torques at hip and trunk areas to control motion but can result in alternations to postural control [8,9]. Although biomechanical changes while carrying excessive load during walking have been extensively studied and findings show that heavier loads may lead to increased trunk, hip and knee flexion and hip and knee extension with greater muscle activation [10], little evidence has been provided regarding the load effects on foot stability during quiet stance [11,12,13]. Quiet or static standing has been used as an assessment of balance performance, representing an individual’s ability to maintain center of pressure (CoP) within the base of support [14]. The CoP reflects the vertical projection of the center of mass (CoM) and an additional torque, which is applied to the ground from the feet [11]. The evaluation of CoP may predict imbalance due to pathogenesis or injury risk [14]. Nowadays, the utility of assessment and data collection from static standing on balance plates may be able to accurately verify and measure the ability and efficiency of human balance [15,16]. However, improved understanding of the biomechanical foot changes in static condition with increased load can provide additional information for special interventions and policies to help minimize the incidence of injury, lost workload days or seeking medical attention [17]. It has been documented that load-induced alternations in postural control are accompanied by compensatory body positions, such as adopting a more forward-leaning trunk posture [18,19] and tilting the pelvis in an anterior direction [19,20]. Recently, a study by Strube et al. [17] showed that external posterior loads of 16 and 25 kg produced changes in mean postural sway velocity, while no significant kinematic adaptations were observed. This would imply that foot characteristics during quiet standing with an additional added mass might be of greater importance for detecting imbalance and body compensations, compared to kinematic outcomes [17]. Similar findings have been obtained previously, where area, speed and excursion of CoP significantly increased with increases in load mass [12,13].

While the effects of excessive load on foot patterns is of extreme interest from a public health perspective, most of the research has been carried out on military personnel [17,18,19,20]. Special police officers are trained to perform tasks and demands in specific environment settings and at a maximal level [21]. Being prepared to execute tactical operations, evidence shows that the equipment carried by special police officers may even exceed the recommended requirements [22]. Since no study has been conducted among special police officers to examine biomechanical changes of foot parameters under different loading conditions, the findings of the present study may be used to establish national protocols to re-position the existing load ergonomically on the body and to prevent future risk of injuries.

Therefore, the main purpose of the study was to examine differences in foot characteristics while standing still under four conditions: (i) ‘without the load’, (ii) ‘a 5 kg load’, (iii) ‘a 25 kg load’, and (iv) ‘a 45 kg load’. We hypothesized that heavier loads would exhibit greater biomechanical foot changes and impaired balance, compared to the ‘no load’ condition.

## 2. Materials and Methods

### 2.1. Study Participants

For the purpose of this cross-sectional study, we randomly selected 30 full-time special police officers, who were part of the Anti-Terrorist Special Police Unit ‘Lučko’ with more than 5 years of service. All participants were healthy and without acute or chronic conditions at the time the study was conducted. All participants were males between 28 and 51 years of age (mean ± SD; age = 40.0 ± 6.0 years, height = 180.0 ± 5.0 cm, weight = 89.0 ± 8.0 kg). All procedures conducted in this study were anonymous and in accordance with the Declaration of Helsinki [23]. The Ethical Committee of the Faculty of Kinesiology and the Anti-Terrorist Special Police Unit ‘Lučko’ approved the study.

### 2.2. Loading Conditions

During testing, each participant walked over the platform with one of four loads: (1) body weight only (‘no load’), (2) with a 5 kg load (‘load 1’, belt + a pistol with a full handgun’s magazine + an additional full handgun’s magazine + a nightstick + handcuffs), (3) with a 25 kg load (‘load 2’, ‘load 1’ + a helmet + a fully equipped backpack + a rifle) and (4) with a 45 kg load (‘load 3’, ‘load 1’ + ‘load 2’ + a bulletproof vest + night vision goggles). The order of the loads was randomized. This equipment represents a standard load in special police officers proposed by the Ministry of Internal Affairs for urban and rural tasks and conditions.

### 2.3. Postural Sway Characteristics

Measurements of all participants were conducted at the same time in the evening hours and at the same place. All respondents were familiar with the measurement proto-col before the measurements. First, the anthropometric characteristics of the examinees were measured, including body height and weight. Ground reaction forces (absolute in N and relative in %) were measured. Each participant stepped on the Zebris medical platform for the measuring of pedobarographic plantar characteristics (type FDM 1.5). The Zebris platform uses 11.264 micro sensors, arranged across the walking area, with a frequency of 300 Hz. It has been used as a diagnostic device for supporting several modes of operation, including static analysis while a participant is standing still [24]. The Zebris platform was connected via USB cable to an external unit (laptop). The data were gathered in real time using WinFDM software for extraction and calculation. Measurement values could be additionally exported in the form of text, picture, and video, while simultaneously comparing the data from both feet. The capacity sensor technology was based on the calibration of every single sensor automatically integrated into a platform. The task was to stand on the platform and maintain a calm position, with arms relaxed by the body and looking straight forward. After 15 sec of measurement, the following parameters were generated: (i) 95% confidence ellipse area (mm^2^), (ii) CoP path length (mm), (iii) CoP average velocity (mm/s), (iv) length of minor axis, (v) length of major axis (mm), and (vi) the angle between Y and major axis (°). For ground reaction forces, the software generated the data for the relative forces distributed under the forefoot and backfoot regions of the foot, as well as for the total foot (%). Of note, the vertical component of the ground reaction forces was collected and analyzed.

### 2.4. Data Analysis

Basic descriptive statistics are presented as mean and standard deviation (SD). The Kolmogorov–Smirnov test was used to assess the normality of the distribution. One-way repeated-measures ANOVA was used to test the effects of load configuration (no load, load 1, load 2 and load 3). Where significant differences between load configurations were observed, a modified Bonferroni procedure was used. All statistical analyses were performed using SPSS v23.0 software (IBM, Armonk, NY, USA) with an alpha level set a priori at *p* < 0.05 to denote statistical significance.

## 3. Results

Basic descriptive statistics of the study participants are presented in Table 1. Changes in static foot characteristics under different loading conditions are shown in Table 2. We identified significant main effects for all static foot variables (*p* < 0.05). Post hoc analysis revealed significant differences between ‘no load’ and the ‘5 kg load’, and the ‘25 kg load’ and the ‘45 kg load’, in CoP path length, average velocity, lengths of minor and major axes and the angle between Y and major axis. For relative forces beneath the forefoot and the backfoot regions, significant differences between ‘no load’ and the ‘5 kg load’, and the ‘25 kg load’ and the ‘45 kg load’, were observed.

## 4. Discussion

The main purpose of the study was to examine differences in foot characteristics during standing while carrying different mass-based equipment. The main findings are: (i) CoP path length, average velocity and lengths of minor and major axes gradually increased with the increased load mass, and (ii) relative forces beneath the left forefoot and the right backfoot decreased, while those under the left backfoot and right forefoot increased with the increased load.

This was the first study that explored the effects of carrying load with different mass on static foot parameters in special police officers. A similar study conducted among military personnel showed an increase in postural sway, where mean postural sway velocity during a double stance increased from 0.27°·s^−1^ to 0.34°·s^−1^ with a 16 kg load and 0.41°·s^−1^ with a 20.5 kg load [17]. With increases in load mass, previous evidence suggests linear increases in the lengths of CoP excursions in the anterior–posterior and the medio-lateral directions, along with increases in plantar motion and the boundary area [12,13]. Compared with ‘no load’, the additional load category used in our study increased CoP length, velocity, and lengths of minor and major axes. Due to biomechanical foot changes and with greater body sway during standing, the body’s center of mass is more likely to approach the boundaries of the base of support, expecting a loss of balance [12]. The greater CoP motion while carrying heavy loads has been discussed previously [25], where an individual maintains an upright stance in the anterior–posterior position by using the ankle and the hip strategy. However, the medial–lateral excursions seem to be of greater importance for stability, because they are directly correlated to a higher likelihood of falls, especially in older individuals [26]. The mechanism of losing postural stability is based on mechanical perspective, where a stable system acts as a kinetic chain between gravity, the base of support and the CoM. When an upright position is affected by external load, the resulting body motion is counterbalanced by one of the strategies which increase postural sway. Indeed, the mechanical approach is often combined with the physiological, where heavier loads carried result in higher demands in terms of heart rate frequency, respiratory changes and proprioceptive systems [27,28]. Unfortunately, we were unable to measure physiological and muscle activation changes while carrying heavier loads, as previous studies have shown that external loads may change the muscle activation patterns necessary to maintain upright stance [28].

Heavy loads are part of military training and specific tasks. However, the increasingly heavy loads create a certain delay in the feedback of the ability to maintain an upright control and posture, which occur with the increased sway away from the equilibrium. To be able not to lose balance, body movement patterns away from equilibrium require corrective adjustments towards the initial position, steadily increasing the structure of the postural sway movements [12]. The greater muscle activity implies greater CoP trajectories when additional mass is added, pointing out that the percentage of activation time should play a significant role in maintaining postural stability.

We also observed changes in relative ground reaction forces during heavy load carriage. It has been well-documented that both vertical and antero-posterior ground reaction forces proportionally increase with heavier load added on the body [10,29]. Recently, a systematic review by Walsh and Low [10] showed that both ground reaction forces and peak plantar pressures increased in loaded conditions compared to unloaded, with the most notable changes in the vertical and antero-posterior directions, but not the medio-lateral direction. However, these findings were based on absolute ground reaction force values, while we observed relative changes (%) beneath forefoot and hindfoot regions of the foot. Although a change and different ratio between forefoot and hindfoot was found in our study, previous evidence has shown no change in the relative distribution of ground reaction forces and pressures on the plantar surface [30]. The discrepancy between the studies comes from different measuring modes, where only studies which measured dynamic gait analysis were included in the review, while we based the findings in static conditions. Additionally, as mentioned in the ‘Introduction’ section, special police officers have different technical and tactical preparations, and equipment being carried, compared to military personnel. Since special police officers bring heavy loads in every mission, it is possible that they develop a motor pattern between excessive load and body position while standing still. This is not surprising, because the average time when the participants were carrying heavy loads was 7.5 h/day after being in tactical missions. Nevertheless, the greater impact forces exacerbated by breaking and propulsive forces may have resulted in the high prevalence of foot blisters (53%) and lower limb stress fractures (47%) in this study. Finally, one possible mechanism of different force distribution beneath foot regions may be explained by the nature of testing, where all participants were instructed to walk on and stand still on the platform, while holding the most natural standing position (parallel to the ground or slightly diagonal stance), which might have led to force increments beneath the left backfoot and the right forefoot region and decreases in the left forefoot and the right backfoot regions. Since this is the first study examining ground reaction forces in special police officers, our findings cannot be comparable to previous ones and future research on this topic is needed.

## 5. Implication for Professional Practice

Carrying heavy loads is a necessity for special police officers. Although excessive loads cause several negative health-related outcomes, including higher injury incidence and lower physical performance [2,3,4], the effort to develop public health strategies and interventions to promote better understanding of equipment positioning and to create a specific design of equipment is still scarce. Greater insight into the effects of carrying load on body biomechanics may be obtained by manipulating the location of the CoM of the backpack by locating weight ‘high’ vs. ‘low’ and ‘close’ vs. ‘away’ from the back position [12]. By using the biomechanical approach, health-related professionals and companies which design police equipment may adequately develop policies which can help in creating and positioning ergonomically appropriate equipment on the body without large negative biomechanical effects or deviations. Therefore, future longitudinal studies conducted among larger sample sizes, adjusted for potential mediators and measured with sophisticated kinematic, kinetic and electromyography systems, should be performed, in order to establish biomechanical changes and proper ergonomic designs of external load.

## 6. Strengths and Limitations

This study has a few strengths. This is the first study conducted among special police officers to establish static foot parameters while carrying different load carriage. Additionally, we used an objective method to collect and generate the data.

This study is not without limitations. First, a cross-sectional design cannot determine causal differences and associations between static foot parameters and different loading conditions. Second, a relatively small sample size (*N* = 30) may have led to insufficient statistical power. Third, no biological and physiological measurements were collected prior to and during the testing (blood samples, heart-rate monitoring, fatigue level and sleep deprivation), which may serve as mediators between static foot parameters and different loading conditions. Fourth, previous studies have used 3D kinematic systems connected with electromyography to assess trunk–hip–knee–ankle positions and muscle activations, which we were unable to measure at the time.

## 7. Conclusions

Static foot parameters, as quantified by traditional CoP measures and generated from the Zebris pedobarographic platform, increased linearly with the increases in external load on the body. The CoP area, average velocity and lengths of minor and major axes rapidly increased with load added, while relative ground reaction forces changed their distribution between forefoot and hindfoot regions of the foot. This study is an addition to the body of literature in examining biomechanical foot characteristics during quiet standing in special police officers.

## Figures and Tables

**Table 1:** Basic descriptive statistics of the study participants (*n* = 30).

Study Variables	Mean (SD)	Min	Max
Age (years)	40.0 (6.0)	27.0	55.0
Height (cm)	180 (5.0)	174.0	189.0
Weight (kg)	89.0 (8.0)	78.4	96.5
Body-mass index (kg/m^2^)	27.5 (1.8)	24.8	30.5
Loading condition			
Load 1 (kg)	5.4 (0.3)	5.1	5.7
Load 2 (kg)	25.6 (2.0)	24.1	27.0
Load 3 (kg)	44.7 (3.2)	42.5	46.9

**Table 2:** Effects of load carriage on postural sway characteristics in special police officers (*n* = 30).

Foot Characteristics	Loading Conditions				Main Effect	
	No Load	5 kg	25 kg	45 kg	*F*-Value	*p*-Value
Time (s)	12.4 ± 1.4 ^a,b,c,d^	16.8 ± 3.3	17.9 ± 3.8	18.4 ± 4.5	19.084	<0.001
95% confidence ellipse area (mm^2^)	160.6 ± 27.2 ^c,e,f^	1298.4 ± 132.4	1609.9 ± 177.1	5169.8 ± 1445.1	8.769	<0.001
COP path length (mm)	127.2 ± 10.4 ^a,b,c,d,e,f^	1029.5 ± 55.3	1307.6 ± 70.0	1367.6 ± 113.8	62.512	<0.001
COP average velocity (mm/s)	10.3 ± 4.4 ^a,b,c,d,e,f^	58.6 ± 16.0	74.4 ± 23.7	78.5 ± 20.0	95.210	<0.001
Length of minor axis (mm)	7.6 ± 3.8 ^a,b,c,d,e,f^	27.8 ± 9.4	29.9 ± 13.8	44.2 ± 14.2	18.541	<0.001
Length of major axis (mm)	23.5 ± 9.5 ^a,b,c,d,e,f^	57.2 ± 15.6	64.7 ± 13.7	105.5 ± 30.4	19.730	<0.001
Angle between Y and major axis (°)	78.1 ± 18.8 ^a,b,c,d,e,f^	75.6 ± 19.3	70.5 ± 21.7	49.3 ± 13.1	9.005	<0.001
Relative force—left forefoot (%)	48.2 ±7.3 ^a,b,c^	39.7 ± 12.1	37.3 ± 18.5	36.3 ± 13.4	4.850	0.003
Relative force—left backfoot (%)	52.1 ± 7.0 ^a,b,c^	60.3 ± 12.1	60.4 ± 16.2	62.7 ± 18.5	3.197	0.026
Relative force—left foot/total (%)	48.0 ± 9.3 ^a,b,c^	53.6 ± 15.8	56.3 ± 11.9	60.2 ± 10.6	5.297	0.002
Relative force—right forefoot (%)	49.8 ± 4.8 ^a,b,c^	60.8 ± 17.9	62.0 ± 22.1	64.1 ± 22.4	3.653	0.015
Relative force—right backfoot (%)	50.2 ± 4.8 ^a,b,c^	39.2 ± 17.9	38.0 ± 22.1	35.9 ± 22.4	3.653	0.015
Relative force—right foot/total (%)	52.0 ± 9.3 ^a,b,c^	46.4 ± 15.8	43.7 ± 11.9	39.8 ± 10.6	5.297	0.002

^a^ denotes significant differences between ‘no load’ and the ‘5 kg load’; ^b^ denotes significant differences between ‘no load’ and the ‘25 kg load’; ^c^ denotes significant differences between ‘no load’ and the ‘45-kg load’; ^d^ denotes significant differences between the ‘5 kg load’ and the ‘25 kg load’; ^e^ denotes significant differences between the ‘5 kg load’ and the ‘45 kg load’; ^f^ denotes significant differences between the ‘25 kg load’ and the ‘45-kg’ load. *p* < 0.05.

## Data Availability

The datasets used and/or analysed during the current study are available from the corresponding author on reasonable request.
